# C-terminal domain of p42 Ebp1 is essential for down regulation of p85 subunit of PI3K, inhibiting tumor growth

**DOI:** 10.1038/srep30626

**Published:** 2016-07-28

**Authors:** Inwoo Hwang, Chung Kwon Kim, Hyo Rim Ko, Kye Won Park, Sung-Woo Cho, Jee-Yin Ahn

**Affiliations:** 1 Department of Molecular Cell Biology, Center for Molecular Medicine, Samsung Biomedical Research Institute, Sungkyunkwan University School of Medicine, Suwon 16419, Korea; 2 Department of Food Science and Biotechnology, Sungkyunkwan University, Suwon 16419, Korea; 3 Department of Biochemistry and Molecular Biology, University of Ulsan, College of Medicine, Seoul 05505, Korea

## Abstract

Potential tumor suppressor p42, ErbB3-binding protein 1 (EBP1) inhibits phosphoinositide 3-kinase (PI3K) activity reducing the p85 regulatory subunit. In this study, we demonstrated that overexpression of p42 promoted not only a reduction of wild type of p85 subunit but also oncogenic mutant forms of p85 which were identified in human cancers. Moreover, we identified the small fragment of C-terminal domain of p42 is sufficient to exhibit tumor suppressing activity of p42-WT, revealing that this small fragment (280–394) of p42 is required for the binding of both HSP70 and CHIP for a degradation of p85. Furthermore, we showed the small fragment of p42 markedly inhibited the tumor growth in mouse xenograft models of brain and breast cancer, resembling tumor suppressing activity of p42. Through identification of the smallest fragment of p42 that is responsible for its tumor suppressor activity, our findings represent a novel approach for targeted therapy of cancers that overexpress PI3K.

Ebp1, an ErbB3 binding protein, is the human homologue of the mouse protein p38-2AG4, which regulates cell proliferation^1^. The gene encoding p38-2AG4, *PA2G4*, possesses three in-frame ATG codons and encodes two alternative spliced isoforms, p48 and p42. p48, which is 54 amino acids longer than p42 at its N-terminus, mediates survival and promotes growth of PC12 cells whereas p42 inhibits cell proliferation and accelerates neurite outgrowth^2^,^3^. The crystal structure of Ebp1 isoforms shows that p42 is missing one-and-a-half helices at the amino terminus^4^, implying that conformational changes associated with the lack of 54 amino acid residues may account for the possible differential roles of these Ebp1 isoforms. In mammalian cells, p48 is the predominant protein whereas p42 is selectively degraded through ubiquitination^5^,^6^.

We have previously suggested the potential oncogenic activity of p48. For instance, we showed that p48 forms a complex with nuclear Akt and prevents apoptosis^2^, and also enhances the kinase activity of Akt^7^. In accordance with its cell growth promoting ability, we demonstrated that p48 is highly expressed in human gliomas and accelerates transformation of cancer cells *in vivo* and *in vitro*, revealing an inverse correlation with poor clinical outcome^6^. p48 associates with the ubiquitin protein ligase HDM2 and promotes the interaction between HDM2 and p53, reducing p53 levels and impairing p53-mediated responses and suggesting that p48 Ebp1 functions as an oncogene by promoting cell survival and proliferation. Moreover, we also demonstrated that p48 sustained Akt-mediated phosphorylation of HDM2 and decreased the reduction of its protein levels by confining HDM2 in the nucleus and preventing HDM2 self-ubiquitination via enhancement of Akt activity.

In contrast to the oncogenic potential of p48, the short isoform of Ebp1, p42, has been considered a tumor suppressor because it binds to the tumor suppressor retinoblastoma protein (Rb), thus inhibiting E2F-1–mediated transcription^8^,^9^, and strongly suppresses both androgen receptor (AR)-mediated transcription and tumorigenesis of prostate cancer cells and salivary adenoid carcinoma cell metastasis in mice^10^,^11^. This is consistent with our observation that p42 Ebp1 suppresses cancerous growth of glioma cells and reduces the size of tumors in glioma mouse models^6^,^12^. Moreover, p42, but not p48, is ubiquitinated and degraded in various human cancer cells, accounting for its weak detection by immunoblotting^5^. In addition, p42 is sumoylated by the TLF/FUS E3 ligase and this sumoylation is important for its antiproliferative effects^13^. More importantly, we proposed p42 as an inhibitor of phosphoinositide 3-kinase (PI3K), a well-known oncogene in various human cancers, through degradation of the p85 regulatory domain of PI3K, and demonstrated that p42 dramatically reduced p85 protein levels by linking p85 to proteasomal degradation mediated by the HSP70/CHIP E3 ligase complex^12^. Collectively, these findings suggest that the shorter isoform of p42 Ebp1 acts as a potential tumor suppressor in various human cancers.

Given that negative regulation of PI3K by p42 through delivery of the HSP70/CHIP complex for p85 subunit degradation accounts for its tumor suppressor function, identification of the smallest fragment of p42 that possesses this activity is worthwhile in order to validate its therapeutic potential. In this study, we describe the minimal functional unit of p42 that efficiently inhibits PI3K through reduction of the p85 subunit and abrogates cancerous growth of tumor cells both *in vitro* and *in vivo*.

## Results

### Reduction of p85 regulatory subunit of PI3K by p42 is essential for antiproliferative activity of p42 in cancer cells

**Figure 1:**
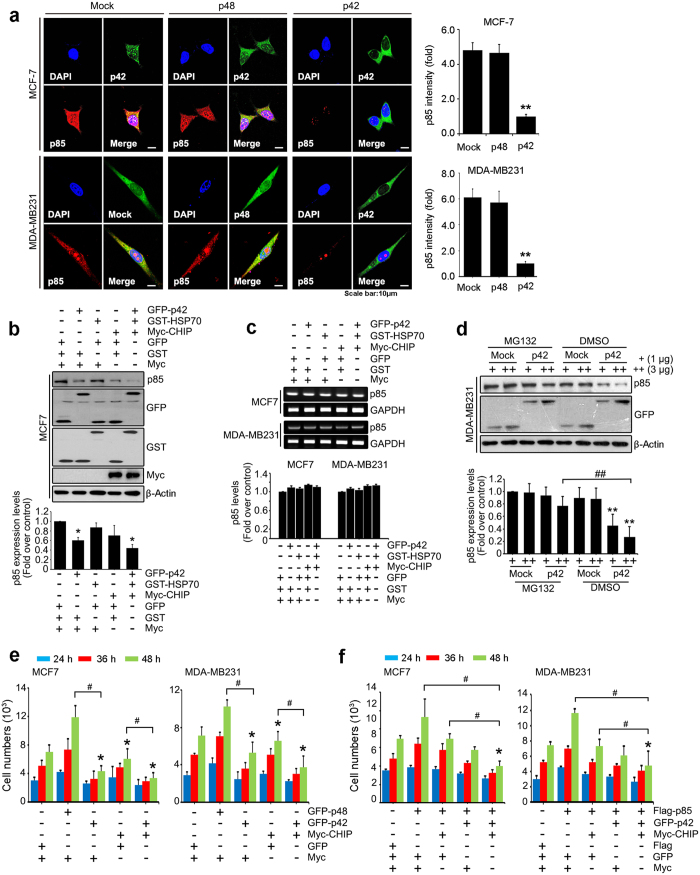
Reduction of p85 regulatory subunit of PI3K by p42 is essential for antiproliferative activity of p42 in cancer cells. (**a**) MCF7 and MDA-MB231 breast cancer cells were transfected with Flag-p85 and GFP-p48, GFP-p42, or GFP-mock vector. At 24 h after transfection, the cells were fixed with 4% paraformaldehyde and immunostained with anti-Flag antibody and Alexa Fluor 594 goat anti-mouse antibody and observed by confocal laser scanning microscopy. p48 or p42 (green), p85 (red) (left) and densitometry analysis of Flag-p85 expression levels (right). ^**^p < 0.005 vs control. (**b**) Endogenous protein level of p85 was measured in MCF7 cells transfected with total 6 μg of plasmids with indicated constructs (for example, for controls vectors (each 2 μg of GFP, Myc, and GST vectors) and for single expression of Myc-CHIP (2 μg of Myc-CHIP with 2 μg of GFP vector and 2 μg of GST vector). Immunoblotting was performed as indicated. ^*^p < 0.05 vs control. (**c**) MCF7 and MDA-MB231 cells were transfected with same strategy with Fig. 1b and after 48 hours, RT-PCR was performed with p85-specific primers. (**d**) GFP- mock vector or GFP-p42 (1 and 3 μg) was transfected into MDA-MB231 cells, following exposure to 10 μM of MG132 for 8 hours. Immunoblotting was conducted as indicated. ^**^p < 0.005 vs control, ^##^*p* < 0.005 vs indicated (**e**) MCF7 (left) and MDA-MB231 (right) cells were transfected (2 × 10^3^ cells per 12 well plate) with total 4 μg of DNA constructs of any combinations. The number of viable cells counted at the indicated times. (**f**) MCF7 (left) and MDA-MB231 (right) cells expressing the indicated constructs (total 6 μg of DNAs with any combinations of different vectors) were plated (2 × 10^3^ cells per 12 well plate) and subjected to proliferation analysis. ^*^p < 0.05 vs control, ^#^*p* < 0.05 vs indicated (1e,f). Values in this figure represent mean ± SEM from three independent experiments and image shown here is representative from at least three independent experiments.

Inhibition of the PI3K pathway has been well characterized as a therapeutic target for the treatment of various human cancers including glioblastoma multiforme (GBM). As we have previously demonstrated that p42 acts tumor suppressor leading to a dramatic reduction in protein levels of the p85 subunit of PI3K in glioblastoma cells and non-small cell lung cancer cells (NSCLC)^12^,^14^, we wondered whether tumor suppressing effects of p42 inducing degradation of p85 is a general event in other types of cancer. In this study we determined that increased p42 expression decreased p85 protein levels in breast cancer cells such as MCF7 and MDA-MB231 cell lines (Fig. 1a). We also confirmed that the p42-mediated reduction in p85 subunit occurs through HSP70/CHIP-dependent proteasomal degradation, exhibiting that co-transfection of HSP70/CHIP with p42 robustly lowers protein levels of p85 subunit but not mRNA levels of p85 (Fig. 1b,c). We also verified any of vector control did not alter p85 protein levels (Supplementary Figure S1). Pretreatment of MG132, a proteasomal inhibitor efficiently protected p85 from p42-mediated degradation (Fig. 1d), supporting that p42 mediated a reduction of p85 protein is ubiquitination-dependent proteasomal degradation. These data indicate that p42-mediated proteasomal degradation of p85 occurs not only in glioblastoma but also in other human cancer cells such as breast cancer cells, and might be a general tumor suppressing mechanism of p42 Ebp1.

Overexpression of p42 inhibits cancer cell proliferation, including that of glioblastoma cells and NSCLC^11^,^12^,^14^. Compared with control vector, ectopic expression of p42 robustly decreased cell proliferation in a time-dependent manner by up to 36.27%, and expression of the E3 ligase CHIP also decreased cell proliferation by ~25.53% after 48 h in MCF7 cells (Fig. 1e left). Co-transfection of p42 with CHIP further enhanced the repressive effect of p42 (Fig. 1e). In contrast, expression of GFP-p48, which acts as oncoprotein in several cancer cells^6^,^14^, increased cell proliferation. Similar result was obtained with MDA-MB231 cells (Fig. 1e right). To determine whether the antiproliferative effect of p42 is resulted from inhibition of the oncogenic stimulating activity of p85, we co-transfected Flag-p85 into MCF7 cells or MDA-MB231 cells with GFP-p42, Myc-CHIP, or GFP-p42/Myc-CHIP. Cells transfected with only Flag-p85 exhibited an enhanced proliferation ratio compared to cells transfected with control vector. However, co-expression of GFP-p42 or Myc-CHIP with Flag-p85 abolished the induction of cell proliferation by p85. Moreover, introduction of GFP-p42/Myc-CHIP into MCF7 or MDA-MB231 cells in the presence of p85 further prevented cell proliferation (Fig. 1f). We also verified none of vector control affect proliferation of both MCF7 and MDA-MB231 cells (Supplementary Figure S2). In addition, depletion of p85 by siRNA abrogated cell proliferation (Supplementary Figure S3) supporting the notion that p42-mediated degradation of p85 is essential for the antiproliferative activity of p42, abrogating oncogenic function of PI3K in cancer cells.

### P42 mediated p85 degradation directed oncogenic mutations of p85 subunit

**Figure 2:**
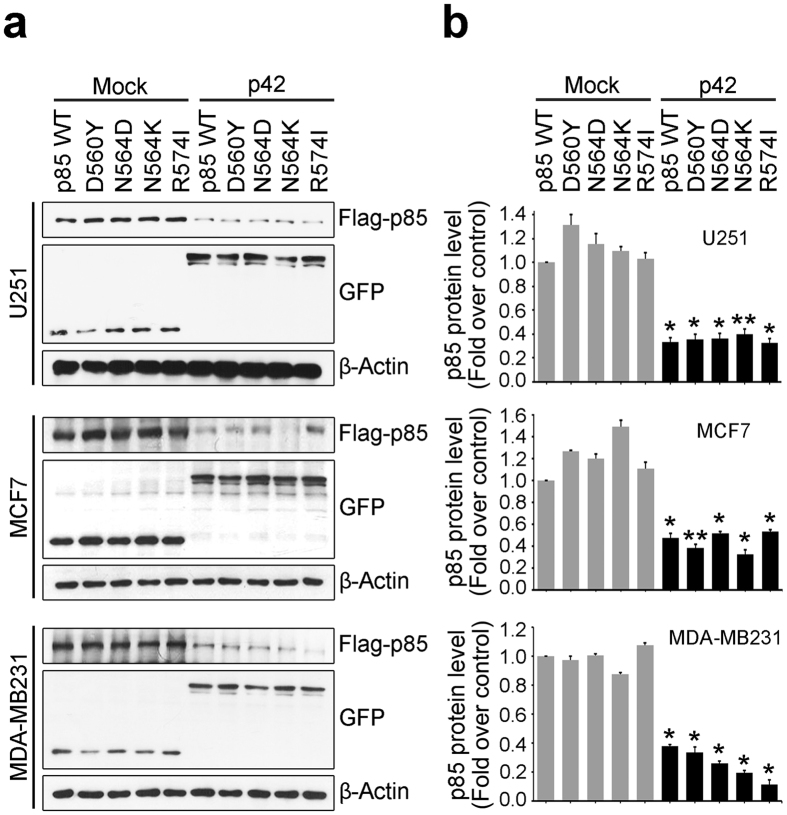
P42 mediated p85 degradation directed oncogenic mutations of p85 subunit. (**a**) Human p85 wild-type or point mutations of the inter-Src homology-2 (iSH2) domain, D560Y, N564D, N564K, and R574I were cloned into pcDNA3 vector (Invitrogen) and co-transfected with GFP-p42 or GFP-mock vector into U251 glioblastoma cancer cells, breast cancer cells, MCF7, MDA-MB231, and incubated in a humidified incubator for 24 h. Protein levels of Flag-p85 wild-type and its mutation forms were detected by immunoblotting against anti-Flag antibody. (**b**) Densitometry analysis of Flag-p85 and its mutations expression levels were shown. A representative blot from three independent experiments is shown for each panel. ^*^p < 0.05 vs control, ^**^p < 0.005 vs control.

The majority of somatic mutations of p85 in various human primary tumors, including glioblastoma, are found in its inter-Src Homology (iSH2) domain and these somatic mutations have been shown to promote the oncogenic function of PI3K *in vivo* and *in vitro*^15^. Because p42 targets the iSH domain for p85 degradation, we wondered whether p42 is able to engage p85 possessing a cancer driver mutation to HSP70/CHIP for degradation. Several oncogenic mutations involving amino acid substitutions in the iSH domain of p85 that were identified in human cancers (D560Y, N564D, N564K, and R574I) were cloned into Flag-tagged vectors and tested to see whether these p85 mutants are regulated by p42 expression. Importantly, p42 expression strongly decreased protein expression of these mutants of p85 to the same extent as expression of p85 WT in breast cancer cells (MCF7 and MDA-MB231) as well as U251 glioblastoma cell (Fig. 2). These results imply that forced expression of p42 might be an efficient therapeutic approach for targeted therapy of PI3K-overexpressing cancers, especially those possessing oncogenic mutations of the p85 subunit.

### The association with both HSP70 and CHIP is essential for p42 mediated p85 degradation

**Figure 3:**
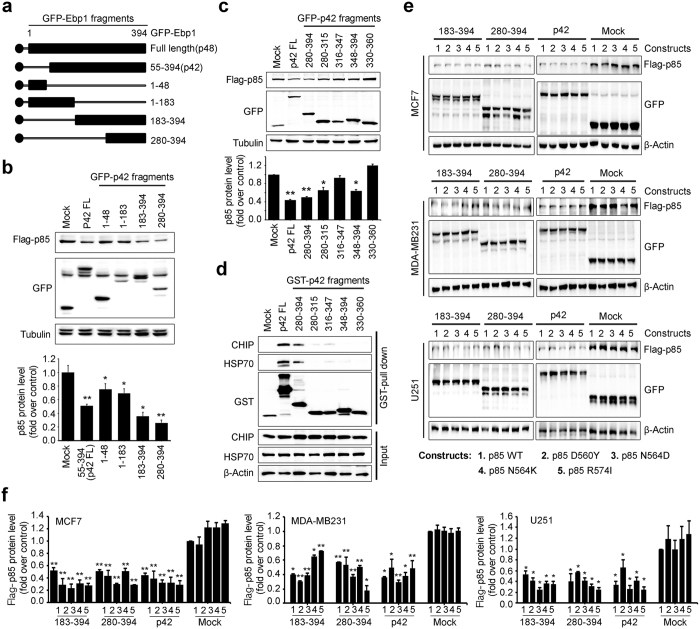
The association with both HSP70 and CHIP is essential for p42 mediated p85 degradation. (**a**) The schematic diagram of the Ebp1 wild-type and its fragments. (**b**) HEK-293T cells were transfected with series GFP-p42 (55–394), p42 fragments (1–48, 1–183, 183–394, and 280–394), GFP-mock vector, along with Flag-p85 and incubated for 24 h. Protein expression levels of Flag-p85 were determined by anti-Flag antibody and a series of GFP-p42 expression was detected by anti-GFP antibody (top). Densitometic analysis of p85 levels were shown in bottom. ^*^p < 0.05 vs control, ^**^p < 0.005 vs control. (**c**) Transfected HEK-293T cells with GFP-p42, its fragments, 280–394, 280–315, 316–347, 348–394, or GFP-mock vector and Flag-p85 were determined Flag-p85 level by immunoblotting with anti-Flag p85 antibody (top) and its quantification is shown (bottom). ^*^p < 0.05 vs control, ^**^p < 0.005 vs control. (**d**) HEK293T cells were transfected with a series GST-p42, GST-p42 fragments, 280–394, 280–315, 316–347, 348–394, 330–360, or control as GST-mock vector. Cell lysates (1 mg) were subjected to a GST pull-down assay for 4 h in the 4 °C cold room and the interaction between endogenous HSP70 and endogenous CHIP with p42 or the fragments were examined by immunoblotting with anti-CHIP antibody, anti-HSP70 antibody. A series of GST-p42 expression was detected by anti-GST antibody. (**e**) Cloned with pcDNA3 vector of Human p85 wild-type or point mutations of the inter-Src homology-2 (iSH2) domain, D560Y, N564D, N564K, and R574I, which are common somatic mutations in PIK3CA in cancers of breast and brain, were co-transfected with GFP-p42, GFP tagged two p42 fragments, 183–394, 280–394 or GFP-mock vector into glioblastoma cells as U251 (bottom) and breast cancer cells as MCF7 (top) and MDA-MB231 (middle). Expression of all the transfected constructs were confirmed, especially Flag-p85 and its mutation forms in the lysates as shown below in each of the panels by immunoblotting with anti-Flag antibody, anti-GFP antibody and its quantification is shown (**f**). A representative blot from three independent experiments is shown for each panel. ^*^p < 0.05 vs control, ^**^p < 0.005 vs control.

To define the minimal functional unit of p42 that mediates p85 degradation, we generated a series of deletion mutants of p42. A schematic representation of the protein domains of p42 is shown in Fig. 3a. Our immunoblotting analysis showed that, among the various GFP-p42 mutant forms, expression of p42 (55–394 amino acids) most strongly diminished p85 protein levels. Interestingly, the inhibitory effect of fragments 183–394 and 280–394 amino acids (aa) was as strong as that of p42. However, overexpression of fragments 1–48, and 1–183 did not elicit p85 degradation compared with p42 (Fig. 3b).

Based on the ability of the C-terminal domain (CTD) 280–394 amino acids to induce a reduction in p85, we further dissected CTD (280–394 aa) to several small fragments to determine whether the C-terminal domain of p42 might provide a tractable molecular target for therapeutic tool design and mechanistic interventions. However, none of the small fragments diminished the expression of p85 protein, whereas p42 WT and the entire CTD (280–394 aa) robustly decreased p85 protein levels (Fig. 3c). Notably, GST-pull down analysis showed that both p42 and the C-terminal domain (280–394 aa) fragment, both of which control p85 protein stability, interacted with the HSP70/CHIP complex, whereas none of the small fragments interacted with the HSP70/CHIP complex (Fig. 3d). These data imply that the ability of p42 to elicit p85 reduction requires both HSP70 and CHIP binding and that the CTD 280–394 fragment is the minimal unit required for p42 activity.

Given that p42 is able to reduce expression of oncogenic mutants of p85 and the CTD (280–394 aa) fragment of p42 is sufficient for p85 degradation, we next tested whether the 280–394 fragment of p42 destabilizes p85 mutant forms. Indeed, the p42-CTD decreased protein levels of p85 mutants containing oncogenic mutations in both breast cancer cells and U251 cells (Fig. 3e). Quantification was analyzed in Fig. 3f. Thus, our data indicate that the p42-CTD (280–394 aa) is both required and sufficient for targeted therapy of PI3K-overexpressing cancers that possess cancer driving mutations in the p85 subunit.

### C-terminal domain of p42 is sufficient to inhibit proliferation, colony formation, and invasion of cancer cells

**Figure 4:**
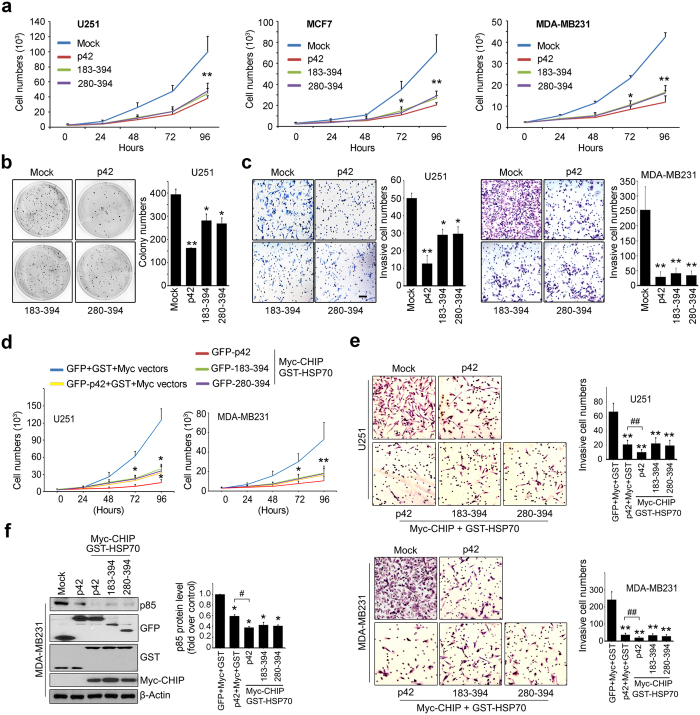
C-terminal domain of p42 is sufficient to inhibit proliferation, colony formation, and invasion of cancer cells. (**a**) U251, MCF7 and MDA-MB231 cells overexpressing GFP-p42, its 183–394, 280–394 fragments, or control were plated (2 × 10^3^ cells per 12 well plate) and viable cells were counted at 0, 24, 48, 72, and 96 h using a disposal hemocytometer. (**b**) Transfected U251 cells (2.5 × 10^3^ cells per 12 well plate) with GFP-p42, GFP- p42 fragments, 183–394, 280–394, or GFP-mock vector were mixed with complete culture media and agarose and seeded on pre-made 0.5% basement agarose plates. Colony forming assay was performed as described in methods and photographed under the light microscope at object x100. Each value represents the mean ± S.E.M. of triplicate measurements. (**c**) U251 (left) and MDA-MB231 (right) cells were transfected with GFP-p42, GFP-p42 fragments or GFP-mock vector and performed *in vitro* invasion assay as described in methods. A representative photograph was provided (objective ×100). Invasive cells were counted in nine random areas. (**d**) U251 and MDA-MB231 cells transfected with total 6 μg of DNA constructs of any combinations of indicated plasmid, were plated (2 × 10^3^ cells per 12 well plate) and proliferating cells were counted at 0, 24, 48, 72 and 96 h. ^*^p < 0.05 vs control, ^**^p < 0.005 vs control (4a–4d). (**e**) U251 (upper) and MDA-MB231 (bottom) cells were transfected with indicated constructs and *in vitro* invasion assay was performed. The cells were photographed (left) and counted in random areas (right). ^**^p < 0.005 vs control, ^##^*p* < 0.005 vs indicated. (f) MDA-MB231 cells transfected with indicated plasmid were subjected to immunoblotting analysis against as indicated antibodies (left) and densitometry quantification is shown (right). ^*^p < 0.05 vs control, ^#^*p* < 0.05 vs indicated. Each value in this Fig. 4 represents the mean ± S.E.M. of triplicate measurements. Scale bar: 200 μm.

Disruption of expression of the proto-oncogene PI3K plays an essential role in a variety of human cancers. Based on the ability of p42-CTD to reduce levels of p85, we next investigated the effects of p42-CTD on cancer cell growth, invasion, and colony formation by transfecting cancer cells with various GFP-p42 fragments. The growth rate assay demonstrated that overexpression of p42 notably suppressed cell proliferation compared with transfection with empty vector, as expected. The inhibition of growth rate by the expression of fragments 183–394 and 280–394 was similar to the suppression of cell proliferation mediated by WT p42 in U251 glioma cells or breast cancer cells (Fig. 4a). To assess the effect of p42 fragments on colony formation we conducted a soft agar assay. After incubation for 3 weeks, cancer cells transfected with p42 WT generated substantially fewer colonies of smaller size compared with cells transfected with control vector. Fragments 183–394 and 280–394 also substantially inhibited colony formation (Fig. 4b left). Quantitative data analysis showed that the p42 fragments suppressed colony formation to approximately 62.7% of that observed with p42 WT (Fig. 4b right). Taken together, our data show that p42 fragments are sufficient for tumor suppressor activity, inhibiting cell growth and transformation to a similar extent as p42 WT.

A previous study showed that transfection of U87MG cells with p48 Ebp1 almost doubled the number of invasive cells compared with control cells. However, p42 reduced cell invasion by about 50%^6^. To explore whether the p42-CTD (280–394 aa) influences invasion of cancer cells to the same extent as p42 WT, we performed an *in vitro* cell invasion assay in Matrigel chambers using U251MG cells or MDA-MB231 cells transfected with GFP-tagged p42 constructs. As expected p42 extensively suppressed cell invasion approximately 80.64% and p42 fragments strongly inhibited cell invasion as much as 70.08% of p42 (Fig. 4c left). Similar result was obtained with MDA-MB231 cells (Fig. 4c right). Thus, overexpression of the CTD fragment (280–394 aa) of p42 is sufficient for inhibition of cell invasion, which is associated with the tumor suppressor activity of p42.

To ascertain tumor suppressing activity of p42 results from CHIP-dependent p42-mediated p85 degradation, we performed cell proliferation analysis and *in vitro* invasion assay in the presence of CHIP/HSP70. Overexpression of p42 only suppressed cell proliferation compared with vector control and co-transfection of CHIP/HSP70 with p42 or its fragments notably decreased growth rate in the both U251 and MDA-MB231 cells (Fig. 4d,e), fitting with our immunoblotting that co-transfection of CHIP with p42 markedly decreased the endogenous p85 level (Fig. 4f), confirming that CHIP is required for p42-mediated p85 degradation.

### C-terminal domain of p42 down-regulates p85 protein stability

**Figure 5:**
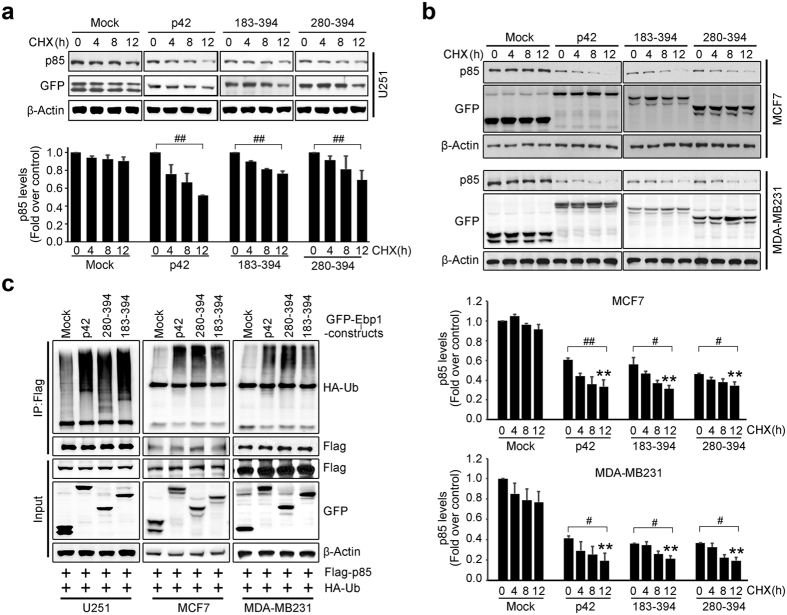
C-terminal domain of p42 disrupts p85 protein stability. (**a**) U251 cells were transfected with GFP-p42, p42 fragments, or control. The cells were incubated in the presence of cycloheximide 10 μg/ml (Sigma, MO, USA) for 0, 4, 8, and 12 h. Endogenous p85 levels were evaluated by immunoblotting (upper) and quantification analysis is shown (bottom). Protein levels of endogenous p85 were measured using a densitometer. Values were normalized to actin and expressed relative to time 0. Each value represents the mean ± S.E.M. of triplicate measurements. ^##^*p* < 0.005 vs indicated. (**b**) Breast cancer cell lines, MCF7 and MDA-MB231, were transfected with GFP-p42, its fragments or GFP-mock vector. The cells were incubated after treat cycloheximide (Sigma, MO, USA) for 0, 4, 8 and 12 h and determined endogenous p85 level by immunoblotting (upper). Quantification analysis is shown (bottom). Each value represents the mean ± S.E.M. of triplicate measurements. ^**^p < 0.005 vs control, ^#^*p* < 0.05 vs indicated, ^##^*p* < 0.005 vs indicated. (**c**) Co-transfected with Flag-p85 and HA-Ub and GFP-p42, GFP-p42 183–394, 280–394, or GFP-Control cells as U251, MCF7 and MDA-MB231 were incubated for 24 h and treated with MG132 (10 μM) for and additional 8 h. The cell lysates (1 mg of protein) were mixed for immunoprecipitation with primary antibody, anti-Flag, with protein A/G beads and incubated for 3 h at 4 °C with gentle agitation and immunoblotting with anti-HA, anti-Flag antibodies. Expression of transfected a serial GFP tagged p42 constructs were confirmed with anti-GFP antibody. A representative blot from three independent experiments is shown for each panel.

We previously found that increased p42 expression dramatically diminished p85 protein levels through controlling p85 protein stability by promoting ubiquitination-dependent proteasomal degradation^12^. To examine whether p42 fragments retain the ability of p42 to mediate specific degradation of the p85 subunit in cells, we determined the half-life of p85 in the presence of various p42 constructs. The half-life of p85 was markedly decreased in cells expressing full-length p42 or p42 fragments compared to control cells whereas p85 levels were not altered by cyclohexaimide (CHX) treatment for up to 8 h in U251 glioma cells, consistent with previous reports that p85 is a relatively stable protein^16^ (Fig. 5a). Similar results were obtained with MCF7 and MDA-MB231 breast cancer cells (Fig. 5b).

To determine whether p42 fragments induce p85 instability through the same molecular mechanism with p42 WT, we examined whether p85 is ubiquitinated in the presence of p42 fragments as it is in the presence of p42 WT in glioma cells and breast cancer cells (Fig. 5c). Expression of p42 fragments (183–394 and 280–394 aa) and p42 WT promoted p85 ubiquitination, suggesting that CTD of p42 is responsible for interaction with the HSP70 and CHIP E3 ligase complex (Fig. 5c), fitting with our observation that at least the 280–394 amino acids of p42 is necessary and required for the formation of a triple complex with HSP70 and CHIP. Thus the CTD (280–394 aa) of p42 facilitates degradation of p85 by the ubiquitin-proteasome pathway.

### Stepwise expression of p42-CTD downregulates p85 protein levels

**Figure 6:**
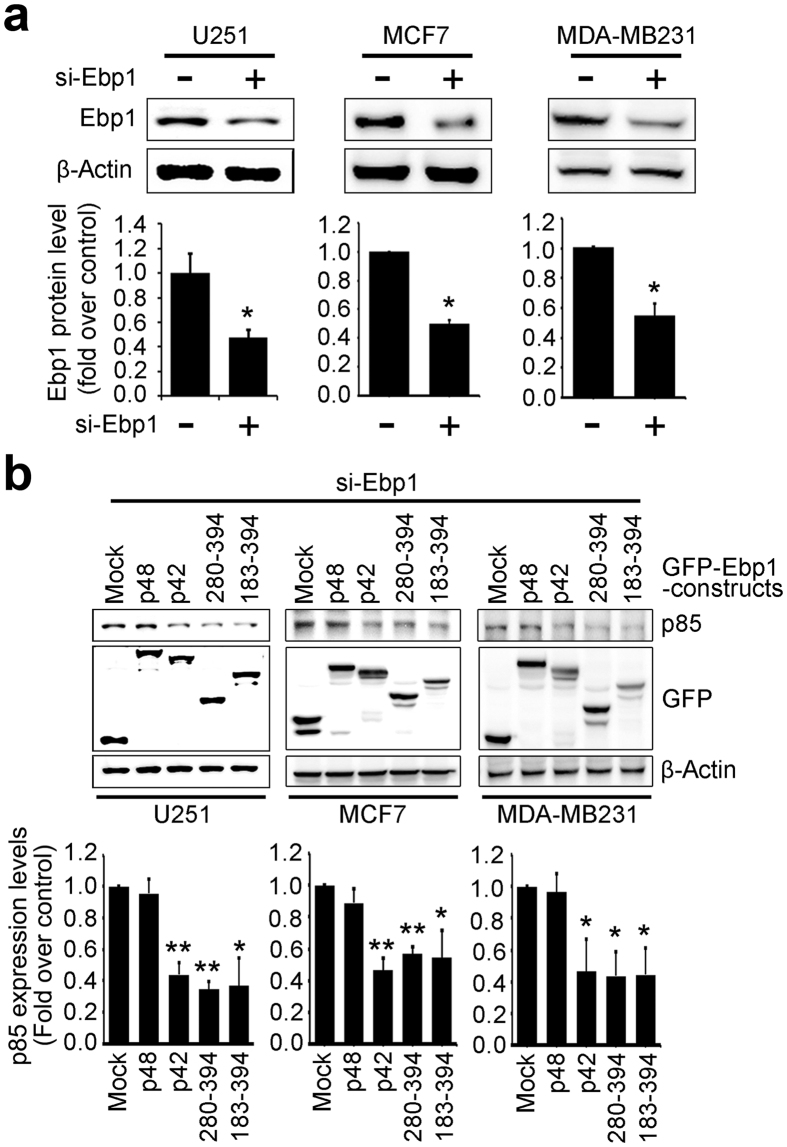
Stepwise expression of p42-CTD down-regulates p85 protein levels. (**a**) Glioblastoma cancer cells, U251, and Breast cancer cells, MCF7 and MDA-MB231, were first transfected with scramble RNA (SCR) or Si-Ebp1. Knockdown of endogenous Ebp1 was analyzed by immunoblotting (upper). Quantification analysis is shown (bottom). ^*^p < 0.05 vs control. (**b**) Depleting endogenous Ebp1 U251, MCF7 and MDA-MB231 cells by si-Ebp1 were transfected with GFP-tagged p48, p42, 183–394, 280–394 or control. After 20 h, endogenous p85 levels were detected by immunoblotting with anti-p85 antibody for the endogenous p85 protein and anti-GFP antibody for the GFP-tagged p42 proteins and analyzed by densitometry analysis. ^*^p < 0.05 vs control, ^**^p < 0.005 vs control. Values in this Figure represent mean ± SEM from three independent experiments and blots shown here are representative from at least three independent experiments.

To evaluate whether p42 fragments are physiologically able to substitute for p42 WT in p85 degradation we depleted endogenous Ebp1 from cells using Si-Ebp1 and then reintroduced various GFP-Ebp1 constructs. Knockdown of Ebp1 was confirmed and quantified by immunoblotting (Fig. 6a) in U251 cells and breast cancer cells. Stepwise expression of p42 after depletion of endogenous Ebp1 provoked approximately 38.14% lower p85 protein levels compared with control (Fig. 6b, second lane). Moreover, C-terminal domain (183–394 and 280–394 aa fragments) of p42 had similar effects to p42 WT, obviously reducing p85 protein levels (Fig. 6b, fourth and fifth lanes). In contrast, exogenous p48 expression following silencing of Ebp1 did not alter p85 protein levels (Fig. 6b, second lane) in U251 cells and MCF7 and MDA-MB231 cells. Quantified data is shown in Fig. 6b bottom. Therefore, the C-terminal domain (183–394 and 280–394 aa fragments) of p42 is sufficient for the tumor suppressor activity of p42 in some breast cancer cells and glioma cells and these fragments appear to exert inhibitory effects by promoting p85 degradation through interaction with HSP70 and CHIP.

### P42-CTD suppresses tumor formation in a mouse xenograft model

**Figure 7:**
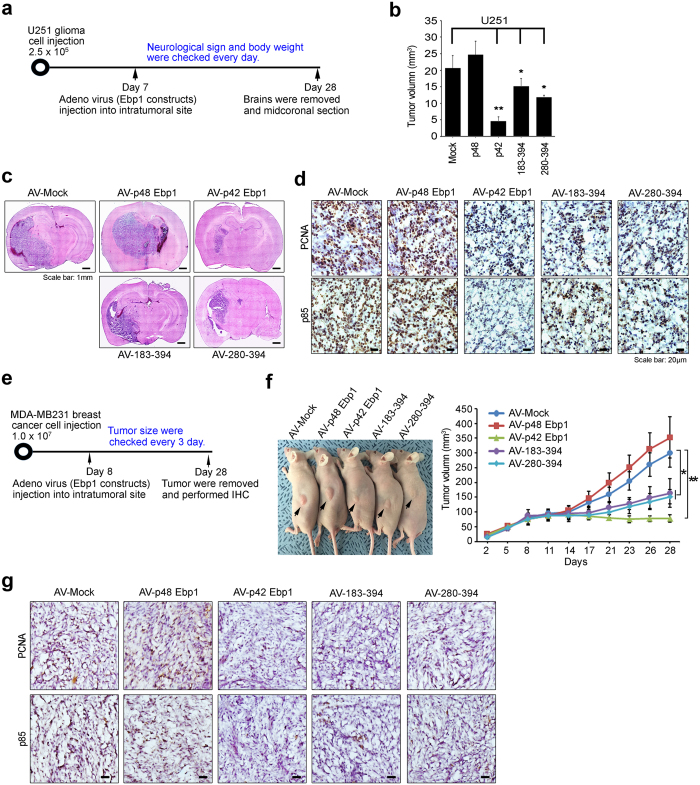
P42-CTD suppresses tumor formation in a mouse xenograft model. (**a**) Timeline demonstrating the intracranial injection of 2.5 × 10^5^ U251 cells followed by the reinjection of adenoviral particles into the same site. (**b**) U251 cells were intracranially injected into mouse brain. On 7 days after tumor implantation, mice received an intratumoral injection of viral particles of adenovirus-GFP, AV-p48, AV-p42, AV-p42-183-394, or AV-p42-280-394. After 21 days, animal were sacrificed and brain removed. The tumor size was measured at the maximal brain tumor dimension after H&E staining of coronal sections. Means ± SEM. ^*^p < 0.05 vs control, ^**^p < 0.005 vs control. (**c**) Representative brain tumors are shown. Scale bar: 1 mm. (**d**) Histological section of U251 brain tumors were stained with PCNA and p85 antibodies. Scale bar: 20 μm. (**e**) Timeline demonstrating the subcutaneous injection of 1.0 × 10^7^ MDA-MB231 cells followed by the reinjection of adenoviral particles into the tumor site. (**f**) The photographs show representative nude mice and tumors (left, arrow indicate subcutaneous tumor area). Animals were monitor up to 30 days and tumor size was measured using calipers at three days intervals (right). The values shown are mean ± SEM. ^*^p < 0.05 vs control, ^**^p < 0.005 vs control. (**g**) Representative tumor images of mice models were stained with anti-PCNA and anti-p85 antibodies. Scale bar: 20 μm.

To examine whether the p42-CTD efficiently exerts tumor suppression activity in glioblastoma carcinogenesis, we established glioma animal models. Briefly, we first generated a mouse xenograft model by intracranial injection of U251 glioma cells into nude mice. After a week of injection for tumor development, we injected the mice with adenovirus (AV) expressing GFP only as control, p48-GFP (positive control), p42-GFP, or two C-terminal domains (183–394 and 280–394 aa) of p42-GFP (Fig. 7a). By 21 days after virus injection, the presence of tumors was evaluated by 8-μm thick serial coronal sections beginning at the inoculation site. Tumor volumes were measured after staining with hematoxylin and eosin. The mean tumor volume of mouse brain injected with p42-expressing AV was dramatically reduced (4.55 ± 1.31 mm^3^ (78% decrease, *p* < 0.001)) compared with that of mice injected with vector only controls (20.64 ± 3.73 mm^3^). Importantly, two C-terminal domains (183–394 and 280–394 aa) of p42 expressing AV was highly reduced tumor volume (15.07 ± 2.46 mm^3^ (27% decrease, *p* = 0.031) or 11.74 ± 0.63 mm^3^ (43% decrease, *p* = 0.013) respectively). As predicted, the average tumor volume for cells expressing p48 was much bigger (24.67 ± 4.08 mm^3^ (19% increase, *p* = 0.042)) than that of control group (Fig. 7b). Representative brain tumors from nude mice are displayed in Fig. 7c. Immunohistochemical analysis of the tumor regions revealed low-intensity p85 expression in the p42-AV and p42 (183–394 and 280–394 aa)-AV injection groups, than that of control or p48-AV injected group, with less frequent expression of proliferating cell nuclear antigen (PCNA) (Fig. 7d).

To further confirm the role of p42-CTD in breast cancer *in vivo* growth, we injected mice subcutaneously with MDA-MB231 breast cancer cells and after tumor development; Eight days after tumor implantation, we injected the mice with adenovirus (AV) expressing GFP only as control, p48-GFP (positive control), p42-GFP, or two C-terminal domains (183–394 and 280–394 aa) of p42-GFP (Fig. 7e). Mice injected with p42-AV or two C-terminal domains (183–394 and 280–394 aa) of p42-GFP after implantation of MDA-MB231 tumor cells developed much smaller tumors than mice injected with the control vector group (Fig. 7f). Immunohistochemical analysis of the tumor regions revealed low intensity p85 and PCNA expression in p42-AV or two of p42-CTD-AV (183–394 and 280–394 aa) injection groups compared with control vector group (Fig. 7g), suggesting that p42 and its CTD inhibits breast cancer growth *in vivo*. Thus our data suggest that the CTD of p42 efficiently inhibits tumor growth *in vivo* by providing a docking site for the association with HSP70/CHIP and thus reducing p85 levels.

## Discussion

The key finding of this report is that in not only glioma cells but also certain types of breast cancer cells, overexpression of p42 permits access of HSP70/CHIP in the vicinity of p85 thus facilitating its proteasomal degradation, as evidenced by the observation that cells expressing p42 displayed a much lower intensity of p85 fluorescence compared with vector or p48-expressing cells. Moreover, we showed that p42 also drives the degradation of p85 possessing driver mutations in the iSH2 domain that are present in patients with cancer. Furthermore, we provide convincing evidence that the C-terminal domain of p42 (fragments 183–394 and 280–394), which are able to bind HSP70/CHIP complex is sufficient for the tumor suppressor activity of p42 in glioma cells and certain breast cancer cells *in vitro* and *in vivo*; our data indicate that fragment 280–394 may be a minimal functional unit of p42 and might have implications for the design of peptide inhibitors of PI3K in the future.

We previously reported that p42 delivers the HSP70/CHIP complex in the vicinity of the p85 subunit thus facilitating immediate degradation of the iSH2 domain, which has been described as having the most oncogenic mutations of p85 and is the location of many reported truncations or deletions^17^,^18^,^19^,^20^. Our current data demonstrated that the C-terminal domain (280–394 aa) of p42 is sufficient to provoke iSH2 domain degradation and exert the tumor suppression activity of p42 (Figs 2, 3 and 5). Our mouse xenograft model strongly supported this notion that C-terminal domain (280–394 aa) of p42 possesses tumor suppressing activity of p42, exhibiting the development of the much smaller tumor with adenovirus that expresses C-terminal domain (280–394 aa) of p42 than mice injected with the control vector (Fig. 7). We have further shown that the p42 fragment (183–394) is responsible for interaction with the p85 subunit^12^ and that the C-terminal domain (280–394 aa) is sufficient for the association with HSP70/CHIP (Fig. 3d), suggesting that the C-terminal domain of p42 might be the minimal functional unit for recruitment of the triple complex of p42-HSP70-CHIP to the p85 subunit, thereby promoting proteasomal degradation of p85.

Although our study showed that p42 and its minimal functional unit, the C-terminal domain (280–394 aa), are able to reduce the protein levels of oncogenic mutant forms of p85 that have been identified in cancer patients (Fig. 3e), it remains to be determined whether any of the oncogenic mutations in the iSH2 domain of p85 prevent p85 degradation by p42-mediated UPS. In this regard it might be interesting to develop small peptides that mimic the biological role of a small fragment of p42 to specifically target the iSH2 domain of p85. Conceivably, targeting this iSH2 domain of p85 instead of directly inhibiting the catalytic domain of p110 of PI3K might result in less severe side effects. The small fragments identified in this study could be used as templates for in silico screening to search for small molecules that share a similar three-dimensional structure as the C-terminal domain of p42, and thus specifically abolish the iSH2 domain to inhibit PI3K activity.

PI3K signaling promotes cell proliferation and increases cell survival, thereby contributing to cancer progression. Moreover, PI3K stimulates tumor progression by promoting cell invasiveness and angiogenesis through its downstream targets^21^,^22^. Thus PI3K inhibition has long been considered a major pharmacological target for drug discovery.

Although ErbB3 is frequently overexpressed in breast cancer and co-expression of ErbB2/3 is known to be a poor prognostic indicator, the role of ErbB3 in breast cancer progression has only recently attempted to pay attention^23^,^24^,^25^,^26^,^27^. As ErbB3 lacks tyrosine kinase activity, its binding partners could be important for biological activity of ErbB3. The p85 subunits of PI3K binds to tyrosine residues of ErbB3 directly and thus provoke intracellular signaling including Akt pathway, which is well known downstream target of PI3K signaling that regulates cell survival and proliferation. Recent study showed treatment of MCF7 and MDA-MB 468 breast cancer cell lines which have low levels of ErbB2 with anti-ErbB3 antibodies decreased cell migration and proliferation along with decreased PI3K and JNK signaling, raising the possibility that ErbB3 might be target in breast cancer patients whose tumors do not overexpress ErbB2^28^. However, ErbB3 is not an easily druggable target due to lack of its tyrosine kinase activity. In current study, we showed that one of ErbB3 binding partner p42 induces degradation of p85 subunit, which is the other ErbB3 binding partner, and subsequently decreased cancerous growth and invasion in MCF7 and MDA-MB231 breast cancer cell lines and notably reduced tumor size in xenograft model. Therefore, upregulation of p42 or inhibition of PI3K by degradation of its p85 regulatory subunit using small fragments of p42 protein not only provides new insights for the development and screening of pharmacologic inhibitors that target PI3K and may serve as positive hits for further drug development for the treatment of cancers but also might be attractive therapeutic approach in certain types of breast cancer.

## Materials and Methods

### Cell culture

HEK293T, U251, MCF7 and MDA-MB231 cells were cultured in Dulbecco’s modified Eagle’s medium supplemented with 10% heat-inactivated fetal bovine serum (FBS) and 100 U of penicillin/streptomycin in a humidified incubator at 37 °C with 5% CO_2_.

### Antibodies, siRNA, and chemicals

Anti-p85 and anti-CHIP acquired from Cell Signaling Technology (Boston, MA, USA). Anti-HSP70 and Ebp1 antibodies were obtained from Abcam (Cambridge, MA, USA). Anti-GFP, anti-Actin, anti-Myc, anti-HA and anti-GST antibodies were acquired from Santa Cruz Biotechnology (Dallas, TX, USA). Anti-Flag antibody was obtained from Sigma-Aldrich (St. Louis, MO, USA). The siRNA specific for Ebp1 (50-UGUAAAUAGUGGUUCUCUGUCCU-GCAU-30), si-p85 #1 (5′-CCAACAACGGUAUGAAUAAUU-3′) and si-p85 #2 (5′-CUGAGUAUCGAGAAAUUGAUU-3′) were obtained from Genolution (Seoul, Republic of Korea). The primer for p85 (5′- CGCTTTCAAACGCTATCTCC-3′, 3′-AGA GCT GGC TGC TGA GAA TC) and GAPDH (5′-ACCACAGTCCATGCCATCAC-3′, 3′-TCCACCACCCTGTTGCTGTA-5′) were obtained from Cosmogenetech (Seoul, Republic of Korea). Cycloheximide was purchased from Duchefa Biochemie (Haarlem, The Netherlands). MG132 was obtained from Sigma (St. Louis, MO, USA). The invasion assay kit (Biocoat Matrigel Invasion Chamber, 354480) was purchased from BD Bioscience. (Franklin Lakes, NJ, USA).

### Constructions of recombinant DNA

Human p85 wild type and the various fragments of p85 were cloned into pcDNA3-Flag. The human p85 point mutations of the inter-Src homology-2 (iSH2) domain, D560Y, N564D, N564K, and R574I were generated by RT-PCR amplification with *in vitro* site-directed mutagenesis system and cloned into pcDNA3 vector (Invitrogen, Carlsbad, CA, USA). The primers for D560Y (5′-TATCGAGAAATTTACAAACGTATGAAC-3′, 3′-GTTCATACGTTTGTAAATTTCTCGATA-5′), N564D (5′- GACAAACGTATGGACAGCATTAAACCA-3′, 3′-TGGTTTAATGCTGTCCATACG-TTTGTC-5′), N564K (5′-GACAAACGTATGAAAAGCATTAAACCA-3′, 3′-TGGTTTAATGCTT-TTCATACGTTTGTC-5′) and R574I (5′-CTTATCCAGCTGATAAAGACGAGAGAC-3′, 3′-GTCTCTCGTCTTTATCAGCTGGATAAG-5′) were synthesized form Cosmogenetech (Seoul, Republic of Korea). HSP70 and E3 ligase CHIP were cloned into pcDNA-GST. Myc-tagged CHIP wild-type and mutation forms, K30R, H260Q, deletion T or U domain, were a gift from Dr. Chin Ha Chung (Seoul National University).

### Western Blotting

Transfected cells were washed with PBS and treated in ice-cold lysis buffer (50 mM Tris-Cl, pH 7.4, 150 mM NaCl, 1 mM EDTA, 0.5% Triton X-100, 1.5 mM Na3VO4, 50 mM sodium fluoride, 10 mM sodium pyrophosphate, 10 mM b-glycerolphosphate, 1 mM phenylmethlysulfonyl fluoride (PMSF) and protease cocktail (Calbiochem, San Diego, CA, USA). Cell extracts were clarified by centrifugation at 14,000 rpm for 10 min. Proteins were denatured and resolved by SDS-PAGE, transferred to Nitrocellulose membrane (Pall Life Science, Ann Arbor, MI, USA). The membranes were blocked in 5% skim milk, and incubated sequentially with primary antibodies and HRP-conjugated secondary anti-bodies^29^.

### Co-immunoprecipitation assay

Cells were rinsed with ice-cold phosphate-buffered saline (PBS) and lysed in buffer (50 mM Tris-Cl, pH 7.4, 150 mM NaCl, 1 mM EDTA, 0.5% Triton X-100, 1.5 mM Na_3_VO_4_, 50 mM sodium fluoride, 10 mM sodium pyrophosphate, 10 mM b-glycerolphosphate, 1 mM phenylmethlysulfonyl fluoride (PMSF) and protease cocktail (Calbiochem, San Diego, CA, USA). The soluble fractions of cell lysates were isolated by centrifugation at 14,000 rpm in a refrigerated microcentrifuge for 10 min. Cell lysates (0.8 to 1 mg of protein) were mixed with primary antibody (anti-Flag, Sigma-Aldrich) with protein A/G beads and incubated for 3 h at 4 °C with gentle agitation. The beads were washed in lysis buffer three times, mixed with 2 × SDS sample buffer, boiled for 10 min, and the sample buffer separated by SDS-PAGE followed by immunoblotting using antibodies.

### GST pull-down assay

Cells were rinsed with PBS and lysed in buffer as described above. Cell lysates (0.5 to 1 mg of protein) were mixed with glutathione-sepharose beads and incubated for 3 h at 4 °C with gentle agitation. The beads were washed in lysis buffer, mixed with 2 × SDS sample buffer, boiled, and analyzed by immunoblotting.

### Ubiquitination assay

U251, MCF7 and MDA-MB231 cells were co**-**transfected with GFP-tagged p48, p42, fragments 183–394 a.a, 280–394 a.a or GFP-mock vector and Flag-p85 and HA-Ub, incubated in a humidified incubator at 37 °C with 5% CO_2_ for 24 h. Cells were treated with MG132 10 μM (Sigma-Aldrich) for 8 h. The cells were washed with PBS and lysed in ice-cold lysis buffer for 30 min. The cell extracts were clarified by refrigerated centrifugation at 14,000 rpm for 10 min and the lysates (1 mg) were mixed with anti-HA antibody with protein A/G beads and incubated for 3 h at 4 °C with gentle agitation. The beads were then spin down at 1000 × g for 1 min and washed three times using the ice-cold lysis buffer. The immunoprecipitated proteins remaining on the beads were boiled in 2 × SDS sample buffer for 10 min, resolved on 8% SDS-PAGE and transferred onto a nitrocellulose membrane. Using anti-HA antibody for HA-Ub and anti-Flag antibody for Flag-p85 to determined ubiquitination of p85.

### Proliferation, invasion, and colony formation assays

U251 and breast cancer cells were transfected with GFP-tagged p48, p42, fragments 183–394 a.a, 280–394 a.a, or GFP-mock vector (2 × 10^3^ cells per 12 well plate) and incubated in a humidified incubator at 37 °C with 5% CO_2_. For the proliferation assay, viable cells were counted on a disposal hemocytometer (In CYTO, DHC-N01-5) at 0, 24, 48, 72, and 96 h^30^,^31^. Invasion assays were performed using a Matrigel invasion assay Kit (BD Bioscience, Inc.). Invasive cells were fixed with 4% paraformaldehyde and stained with 1% Crystal violet^32^. For the colony-forming assay, cells were seeded on 6-well plates in 0.35% agarose (supplemented with complete medium; BD Bioscience) and cultured for 4 weeks. The cells were fixed with 4% paraformaldehyde, stained with crystal violet, and the colonies were counted.

### Immunofluorescence

Transfected U251, MCF7 and MDA-MB231 cells were seeded in poly-l-lysine coated glass coverslips in 24 well tissue culture plates. 24 h later, the slides were fixed in 4% paraformaldehyde for 15 minutes at room temperature, permeabilized in PBS containing 0.25% Triton X-100 for 10 minutes, and blocked in 2% BSA for 30 min^33^. The slides were rinsed twice with PBS, and the slides were immunostained using primary antibodies in 1% BSA in PBS for overnight at 4 °C cold room, rinsed four times with PBS, incubated with secondary antibody produced Alexa Fluor 594 goat anti-mouse (Invitrogen, Rockford, USA) for 1 h at room temperature. Nuclei were counterstained with Hoechst, and washed four times with PBS. The Slides were mounted on microscope sliders, using VECTASHIELD^®^ Mounting Medium (Vector Laboratories, United States). Immunostained images were acquired using a laser scanning confocal microscope LSM 710 META Duoscan (Carl Zeiss, Germany) equipped with a 40 × water immersion C-Apochromat objective (Carl Zeiss) at room temperature (22 °C). The confocal microscope was controlled using LSM software version 4.2 (Carl Zeiss). Confocal microscopy was performed in the Research Core facility, SBRI.

### Mouse xenograft model and Immunohistochemistry

This study was reviewed and approved by the Institutional Animal Care and Use Committee (IACUC) of Sungkyunkwan University School of Medicine (SUSM) (code16-23). SUSM is an Association for Assessment and Accreditation of Laboratory Animal Care International (AAALAC International; No. 001004) accredited facility and abide by the Institute of Laboratory Animal Resources (ILAR) guide. All experimental procedures were carried out in accordance with the regulations of the IACUC guideline of Sungkyunkwan University. Briefly, 6- to 8-week-old athymic nude (nu/nu) mice were housed in laminar-flow cabinets under specific pathogen-free conditions.For the establishment of glioma model, animals were anesthetized by intraperitoneal (i.p.) injection with 12 mg/kg of xylazine (Rompun; Cutter Laboratories, Shawnee, KS, USA) and 30 mg/kg of ketamine (Yuhan Ketamine; Yuhan Co., Ltd, Seoul, Korea) and were held in a stereotactic frame with an ear bar. Human glioblastoma U251 cells (2.5 × 10^5^ cells in a volume of 2.5 μl of PBS) were slowly injected 3-mm deep into the brain with a Hamilton syringe. A week days after tumor implantation, the animals were randomly divided into five groups (n = 5 per group) and reinjected viral particles expressing adenovirus (AV)-GFP, AV-p48, AV-p42, AV-p42-183-394 and AV-p42-280-394 (10^11^GC/ml) into the same site. At 21 days after viral infection, tumors were removed from the sacrificed mice, fixed in 10% buffered formalin solution, and frozen in OCT compound. Frozen tissues were cut coronally at the injection site and section into 8-μm thick slices. Coronal sections of the whole tumors were processed for histological and immunohistochemical analyses. Immunohistochemical stains were applied using antibodes against PCNA and p85. Images were acquired using an inverted Zeiss microscope. MDA-MB231 cells (1 × 10^7^ cells in matrigel) were injected subcutaneously into the right flank of each nude mouse (total 20 mice). Eight days after implantation, animals were randomized into five groups (n = 4 per each group) when the solid tumor size reached 5 mm diameter. Then, 0.5 μl of viral particles expressing adenovirus (AV)-GFP, AV-p48, AV-p42, AV-p42-183-394 and AV-p42-280-394 were administrated to tumor region. The tumor size was monitored every three days using calipers, and tumor volume (V) was calculated using the formula V = (*ab*)/2, in which *a* is the longest and *b* is the shortest diameter of the tumor. After 20 days of AV injection, animal tumors were removed and frozen sections of the whole tumors were processed for histological and immunohistochemical analyses. Representative tumor of AV-GFP, AV-p48, AV-p42, AV-p42-183-394 and AV-p42-280-394 animals was stained with an anti-PCNA antibody (proliferative cells) and P85 expression was determined by staining with an anti-p85 antibody. Arrows indicate the PCNA-positive cells and p85-positive cells. Scale bar = 20 μm.

### Statistical Analysis

Data are expressed as mean ± SEM of triplicate measurements from three independent experiments. Statistical analysis was performed using Sigmaplot Statistical Analysis Software (Systat software). All studies were performed in a blinded manner. Statistical significance was defined as ^*^p < 0.05, ^**^p < 0.005, ^#^*p* < 0.05, ^##^*p* < 0.005.

## Additional Information

**How to cite this article**: Hwang, I. *et al*. C-terminal domain of p42 Ebp1 is essential for down regulation of p85 subunit of PI3K, inhibiting tumor growth. *Sci. Rep.* **6**, 30626; doi: 10.1038/srep30626 (2016).

## Supplementary Material

Supplementary Information
