# Supplementary material for: C-terminal domain of p42 Ebp1 is essential for down regulation of p85 subunit of PI3K, inhibiting tumor growth
Source: Sci Rep. 2016 Jul 28;6:30626. doi: 10.1038/srep30626 (PMC4964336; doi:10.1038/srep30626)
Supplement: Supplementary Information [file srep30626-s1.pdf]

Supplementary Information

C-terminal domain of p42 Ebp1 is essential for down regulation of p85 subunit of PI3K, inhibiting tumor growth

Inwoo Hwang[1], Chung Kwon Kim[1], Hyo Rim Ko[1], Kye Won Park[2], Sung-Woo Cho[3] and Jee-Yin Ahn[1]*

[1]Department of Molecular Cell Biology, Center for Molecular Medicine, Samsung Biomedical Research Institute, Sungkyunkwan University School of Medicine, Suwon 16419,Korea.

[2]Department of Food Science and Biotechnology, Sungkyunkwan University, Suwon 16419, Korea.

[3]Department of Biochemistry and Molecular Biology, University of Ulsan, College of Medicine, Seoul 05505,Korea.

***Correspondence should be addressed to:** Jee-Yin Ahn, Department of Molecular Cell Biology, Sungkyunkwan University School of Medicine, 2066, Seobu-ro, Jangan-gu, Suwon 16419, Korea. Phone: 82-31-299-6134; Fax: 82-31-299-6139; E-mail: jeeahn@skku.edu

Supplementary Figures

Supplementary Figure S1

Any of vector control did not alter protein levels of p85 subunit of PI3K

MCF7 cells were transfected 4 μg of each control vectors (GFP, Myc or GST vector) respectively and determined endogenous p85 protein levels by immunoblotting with anti-p85, GFP and GST antibodies (left). Densitometry analysis of p85 expression levels were shown (right). A representative blot from three independent experiments is shown for each panel.

Supplementary Figure S2

None of vector control affects proliferation of breast cancer cells

MCF7 and MDA-MB231 were transfected with 4 μg of each control vectors (GFP, Flag or Myc vector). Transfected cells and non-transfected control cells were plated (2 x 10^3 cells per 12 well plate). The viable cells were counted at 24, 36 and 48 h by disposal hemocytometer. Values in this figure represent mean ± SEM from three independent experiments and image shown here is representative from at least three independent experiments.

Supplementary Figure S3

Depletion of p85 subunit of PI3K by siRNA inhibited cell proliferation in glioma and breast cancer cells.

(a) U251 and (b) MDA-MB231 cells were transfected with scramble RNA(SCR), p85 targeted siRNA #1 and #2 (p85 #1, p85#2), and subjected to immunoblotting for p85, p-AKT(S473), total AKT and Actin (left). MTT assay was performed to estimate the cell proliferation of U251 or MDA-MB231 cells with SCR and knock-down of p85 by siRNA (right). All data represents the means ± SEM of at three independent experiments. *$p < 0.05$ vs control.

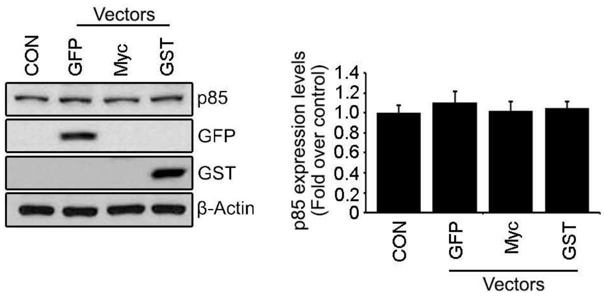
Vectors
CON
GFP
Myc
GST
p85
GFP
GST
β-Actin
p85 expression levels
(Fold over control)
1.4
1.2
1.0
0.8
0.6
0.4
0.2
0
CON
GFP
Myc
GST
Vectors

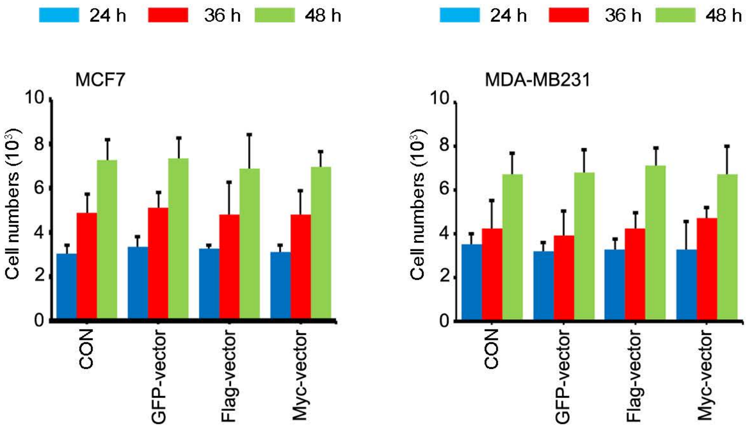
24 h
36 h
48 h
MCF7
MDA-MB231
Cell numbers (10^3)
0
2
4
6
8
10
CON
GFP-vector
Flag-vector
Myc-vector

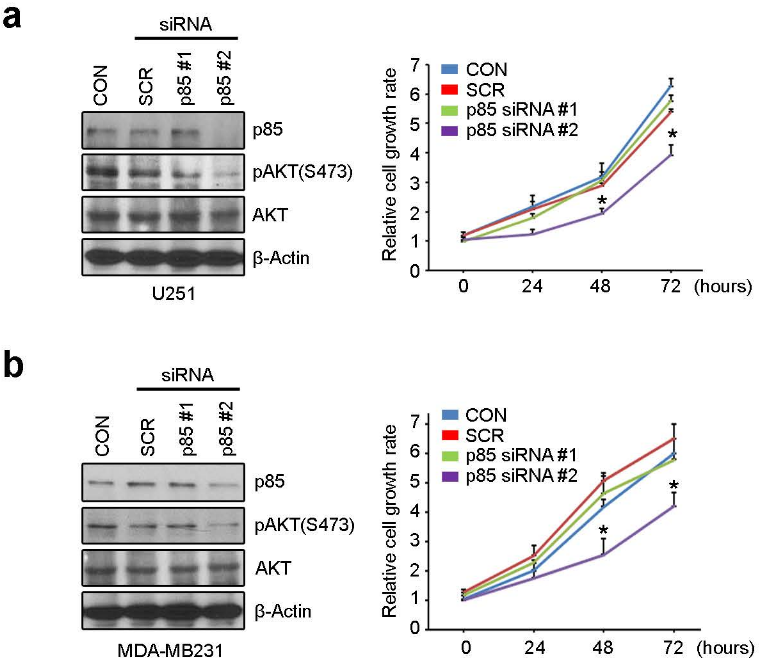
a
siRNA
CON
SCR
p85 #1
p85 #2
p85
pAKT(S473)
AKT
β-Actin
U251
Relative cell growth rate
CON
SCR
p85 siRNA #1
p85 siRNA #2
0
24
48
72
(hours)
b
siRNA
CON
SCR
p85 #1
p85 #2
p85
pAKT(S473)
AKT
β-Actin
MDA-MB231
Relative cell growth rate
CON
SCR
p85 siRNA #1
p85 siRNA #2
0
24
48
72
(hours)
